# Estimating Fuel Moisture in Grasslands Using UAV-Mounted Infrared and Visible Light Sensors

**DOI:** 10.3390/s21196350

**Published:** 2021-09-23

**Authors:** Nastassia Barber, Ernesto Alvarado, Van R. Kane, William E. Mell, L. Monika Moskal

**Affiliations:** 1 Forest Resilience Laboratory, School of Environmental and Forest Resources, College of the Environment, University of Washington, Seattle, WA 98195, USA; alvarado@uw.edu (E.A.); vkane@uw.edu (V.R.K.); lmmoskal@uw.edu (L.M.M.); 2 Pacific Northwest Research Station, USDA Forest Service, Portland, OR 97204, USA; william.mell@usda.gov

**Keywords:** unmanned aerial vehicle, fuel moisture content, wildfire, grassland

## Abstract

Predicting wildfire behavior is a complex task that has historically relied on empirical models. Physics-based fire models could improve predictions and have broad applicability, but these models require more detailed inputs, including spatially explicit estimates of fuel characteristics. One of the most critical of these characteristics is fuel moisture. Obtaining moisture measurements with traditional destructive sampling techniques can be prohibitively time-consuming and extremely limited in spatial resolution. This study seeks to assess how effectively moisture in grasses can be estimated using reflectance in six wavelengths in the visible and infrared ranges. One hundred twenty 1 m-square field samples were collected in a western Washington grassland as well as overhead imagery in six wavelengths for the same area. Predictive models of vegetation moisture using existing vegetation indices and components from principal component analysis of the wavelengths were generated and compared. The best model, a linear model based on principal components and biomass, showed modest predictive power (r² = 0.45). This model performed better for the plots with both dominant grass species pooled than it did for each species individually. The presence of this correlation, especially given the limited moisture range of this study, suggests that further research using samples across the entire fire season could potentially produce effective models for estimating moisture in this type of ecosystem using unmanned aerial vehicles, even when more than one major species of grass is present. This approach would be a fast and flexible approach compared to traditional moisture measurements.

## 1. Introduction

Existing research on wildfire behavior is vast and has a broad range of motivations, including biodiversity concerns, carbon sequestration, firefighting, health risks posed by smoke, and danger to homes in the Wildland Urban Interface (WUI) [1,2,3]. The prevalence and severity of fires have measurably increased since 1960 due to increasing average temperatures, severe winds, and land-use changes [4,5,6]. The number of homes in the WUI is also increasing. This increase provides significant motivation for further study of fire behavior, especially in ecosystems with increased WUI development [7,8]. In addition to determining the risk to homeowners, an understanding of fire behavior and the factors that contribute to ignition of wildland fuels is desirable in order to better inform management decisions, such as prescribed burn planning or fuel thinning, to reduce the risk of fire [9,10,11].

It is not only temperature increases, but also decreases in relative humidity, that contribute to increased fire frequency and severity [12]. Fuel moisture, which responds to these climate effects, can be prohibitively time-consuming to measure using conventional methods [13]. However, moisture is an important factor in predicting fire risk and behavior and is necessary for informed land management decision-making [14,15,16,17]. The importance of accurately measuring fuel moisture only increases as climate change becomes a dominant factor in fire behavior and as researchers develop models that make predictions based on fluid dynamics.

Two technological developments are occurring that can increase the ease and accuracy of predicting fire behavior and evaluating risk. One of these is the increased availability of computational resources and software. This includes both processing software/techniques and computational power [18]. The other is the increased availability of aerial imagery from satellite data, airplanes, or unmanned aerial vehicles (UAVs), which are becoming increasingly affordable and user-friendly [19,20]. These two developments make it possible to create models more informed by observational data, notably moisture data.

Historically, fire models have been empirical or semi-empirical and do not explicitly simulate the processes that drive fire behavior. Examples of these models include the Rothermel model [21] and the Balbi model [22]. The Wildland–Urban Interface Fire Dynamics Simulator (WFDS) model is an extension of the FDS model created by the National Institute of Standards and Technology. It is a physics-based model that aims to improve general understanding of fire-behavior in a more broadly applicable way than models which rely on very species-specific measurements [2,23]. Its development will support an understanding of fire behavior and ultimately inform and improve the performance of empirical models, especially in complex situations, such as WUI areas with a combination of roads, vegetation, and buildings [8]. WFDS is different from most existing models in that it integrates fuel conditions, atmospheric effects, and fire physics into a model of the physical processes at work [24]. In order to validate the WFDS model, as well as many other models with regards to moisture inputs, methods are needed to create spatially explicit moisture measurements [25].

Researchers usually study fuel moisture in two separate components: live (LFMC) and dead (DFMC) moisture content. In addition to variation due to fuel type and size, dead vegetation, such as branches, fallen leaves, and duff, responds more immediately to atmospheric changes, while live fuel responds more to soil moisture, plant physiology, and longer-term weather trends [15]. Therefore, it makes sense to model them separately. FMC calculation in the field involves measuring the weight of the fuel before and after oven drying [26].

Reflectance data in a variety of wavelengths have shown promise in detecting live vegetation moisture. One way to obtain specific information about the spectral attributes of vegetation is to measure specific wavelengths in relatively narrow bands so that the effects that change the reflectance in a given band can be isolated [27]. Many physical effects contribute to the spectral signature of vegetation, but the most important is energy changes in covalent bonds [28]. Chlorophyll, the primary material in grass that reflects light, can undergo spectral changes due to plant stress in different environmental conditions, but which are highly dependent on species. A significant amount of research exists using imagery from satellites, such as MODIS [29,30,31], from other airborne sensors such as AVIRIS, or from UAV-mounted sensors [1,32]. While much of the existing research relies on the Normalized Difference Vegetation Index (NDVI), an index using visible and near-infrared wavelengths, some research has shown indices using short wave infrared (SWIR) are also a good predictor of vegetation moisture [33,34,35]. In some ecosystems, an index based on visible wavelengths alone, the Visual Atmospheric Resistance Index (VARI) performs better than NDVI [36,37]. Table 1 shows a summary of these indices. With so many sensor options, large differences in model performance between ecosystems, and a broad range of sensor prices, it is important to determine for a given ecosystem which techniques work best.

Many studies use satellite data to predict fuel moisture in a variety of ecosystems, including shrublands and chapparal [30,38] as well as grasses [32,34], and different vegetation indices perform best in different ecosystems. Comparatively few studies exist which measure the moisture of non-agricultural vegetation using UAV imagery. This study explores the use of two different UAV-mounted sensors recording spectral information in visible and infrared wavelengths for deriving high spatial resolution fuel moisture inputs for fire behavior models. This method is evaluated using traditional field measurements as a “ground truth” and builds a predictive model based on reflectance values in six wavelength bands. In this way, recommendations can be made for future research in live fuel moisture estimation.

## 2. Materials and Methods

### 2.1. Site Selection

**Figure 1:**
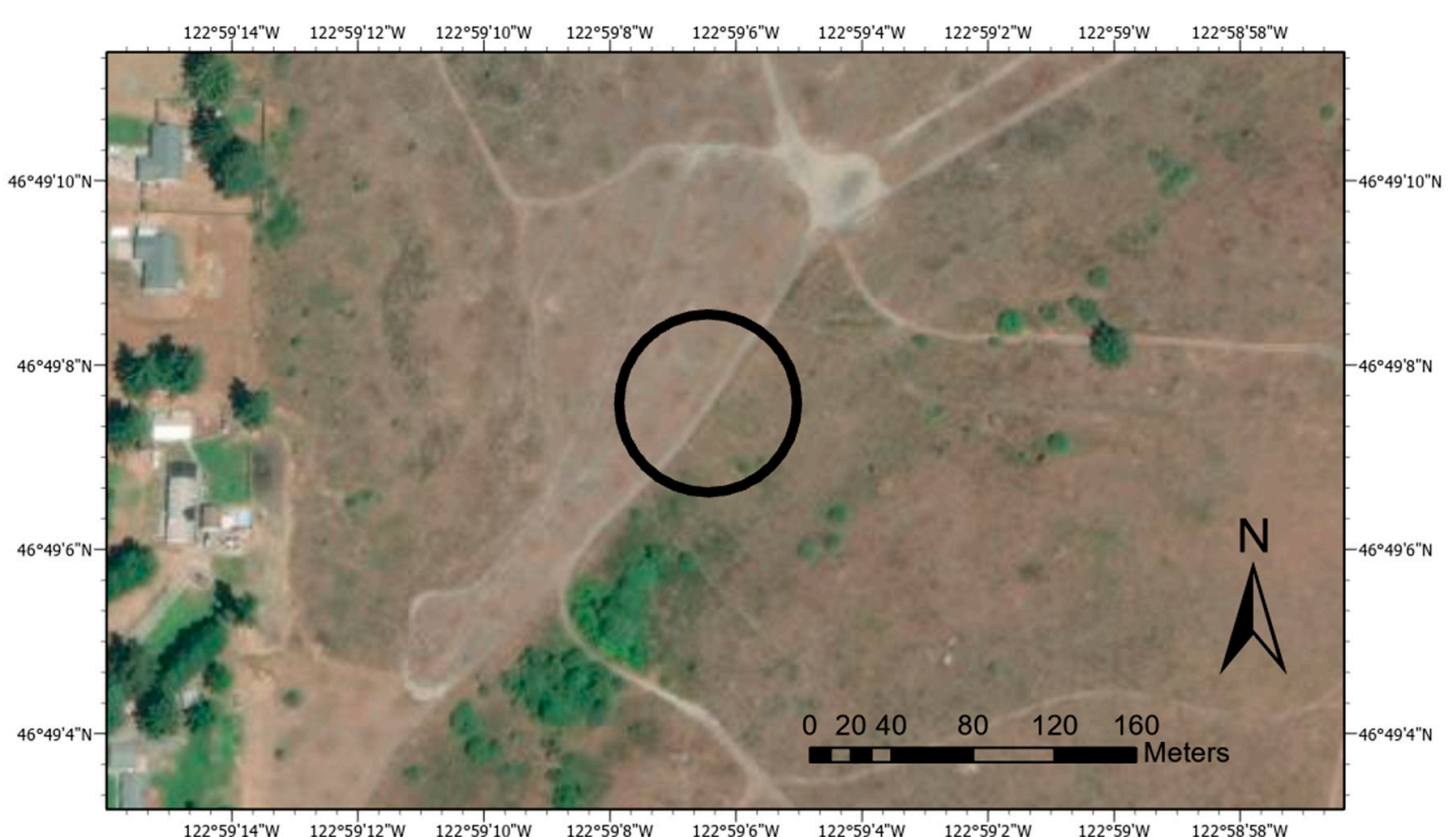
Overhead Image of Study Site in Context, with the Study Area Circled.

The study area (Figure 1) was in western Washington, USA at the Center for Natural Lands Management (CNLM) Mazama Meadows land holding in Rochester, WA. The area included 120 vegetation sample plots, each 1 m × 1 m, arranged in a grid. There were sixty plots on each side of a dirt road. The vegetation on one side of the road was taller and primarily consisted of tall oat grass (*Arrhenatherum elatius*). In contrast, the vegetation on the other side if the road was more mixed, including the oat grass as well as other grasses such as *Agrostis stolonifera*. These two types of vegetation are referred to as “tall” and “short”, respectively. Both vegetation types included a small amount of scotch broom (*Cytisus scoparius*), which was expected to have a far higher water content than the grass fuels. The fuels ranged in height from approximately 0.1 m to 1.5 m. The site was appropriate for this study because it was free of trees or other obstacles that could create shadows, dominated by grass species in a fire-prone WUI area, and relatively flat and uniform to avoid effects from topography. This site is typical in many ways of a WUI habitat that is fire-prone and for which fire modeling is important because of the danger to nearby buildings and residents. For this reason, sites such as this (including this site) are likely what will be used in the future to validate the predictions of the WFDS model.

For moisture data, timing is also critical. A highly accessible site was chosen to allow for development of the data collection process over the season, instead of one long visit to a remote area. The site also has many sunny days in the summer, which enabled the collection of UAV imagery and field data as close as possible to solar noon and in direct sunlight. This was necessary to create consistent lighting conditions for comparison between flights. The data collection was performed on 22 September 2020. The two flights were at 9:34 a.m. and 2:56 p.m., and the field data for each were collected within two hours after the start of the flight.

Figure 1 shows the study site in context, with visibly different types of vegetation on either side of the primitive road.

### 2.2. Imagery Data Collection

The data was collected using a FLIR Tau SWIR camera and the Micasense RedEdge multispectral camera mounted to a Da-Jiang Innovations (DJI) M600 drone. The Tau is a specialized video camera that records video in a single, short-wave infrared band. It was equipped with a 25 mm lens. The Micasense is a five-band camera that records separate images in the red, green, blue, red edge, and near-infrared (NIR) bands. The Micasense is a product designed for non-engineers and is comparatively inexpensive and easy to use. The Tau was controlled and powered by an onboard Raspberry Pi, and the Micasense was controlled by a mobile app over its WiFi network. Both the Micasense and the Raspberry Pi were powered using battery packs mounted to the drone.

A resolution of 5 cm (cm) or less was desirable to ensure the variation within each plot was captured. The following formulas provide the horizontal and vertical angle of view (*AOV*) and the camera coverage, where *p* is the sensor size in that dimension, *d* is the distance of the camera from the subject, and *f* is the focal length [39].

Equation (1): Resolution Calculations.

(1) $$\begin{array}{c} AOV=2*\arctan\left(\frac{p}{2f}\right)\\ C=2*d*\;\tan\left(\frac{AOV}{2}\right) \end{array}$$

The Tau, which was the limiting factor for both resolution and coverage, has a 9.6 mm × 7.68 mm sensor; so, with a 25 mm lens and a typical flight height of 16 m, this gave a coverage of 6.1 m horizontally and 4.6 m vertically. This corresponds to a resolution of 1.1 cm.

UgCS was chosen as the flight control software due to its ability to set specific ground speed and elevation for repeatable flights. UgCS also includes functionality to determine image overlap in flight planning. Though there is some variation among previous studies, a frequent target for front overlap is 60–85% or higher and 40–85% for side overlap. In this study the side and front overlap were both set to 85% based on Micasense parameters to improve tie point identification [40,41,42]. These parameters successfully produced images with 85%+ overlap or greater for the Micasense images, but the side overlap was less for the Tau images. The UAV flew at a height of 16 m and a speed of 3 m/s. A double-grid pattern was used to ensure proper coverage of the study area. Each flight took approximately 10 min. 

**Figure 2:**
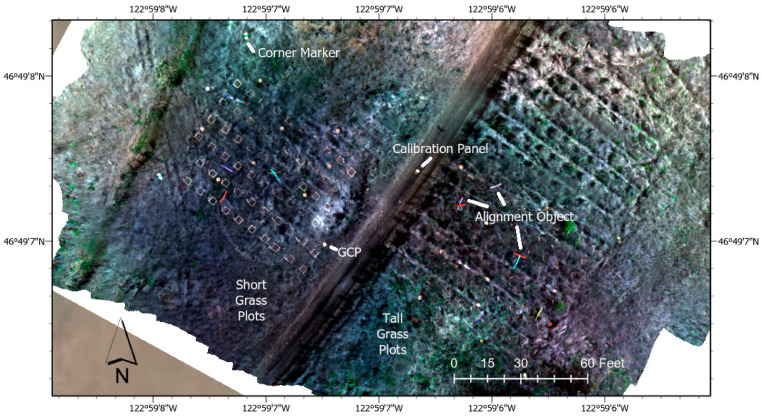
Layout of Study Area Including Alignment and Calibration Objects.

Foam pool noodles and folding pieces of sheet metal were placed around the study area in addition to the ground control points (GCPs) to be visible from above and aid in image alignment as shown in Figure 2. The drone was also flown over the Micasense calibrated reflectance panel to obtain calibration data from known reflectance values.

Two flights were flown, and half of the field data was collected (randomly selected plots) after each flight. This helped make it possible for the field data to be collected closer to the same time as the imagery data, as the field data collection process was time-consuming.

Table 2 shows the specific wavelengths recorded in the study, along with the commonly used MODIS wavelength bands nearest to them for comparison.

### 2.3. Field Data Collection

**Figure 3:**
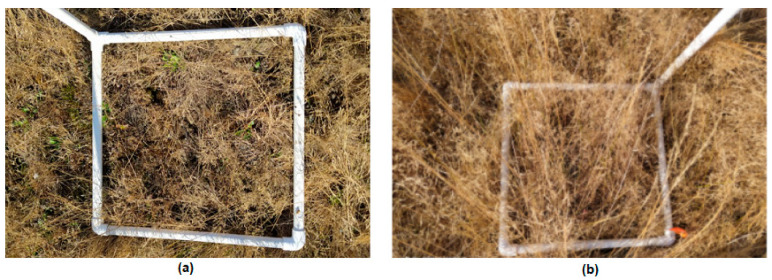
Examples of Plots in Short Grass (**a**) and Tall Grass (**b**).

The plots were destructively sampled, placing the vegetation in a sealed plastic bag, and samples were weighed before and after 48 h of oven drying at 70 degrees Celsius. Each plot was determined by a 1 m × 1 m area of PVC pipe and a vertical pipe labeled with the plot number, as shown in Figure 3. The vertical pipe made it much easier to locate the plots in the tall grass in overhead imagery, and it also allowed the field crew to find their assigned plots much more quickly. Before sample collection, the field technicians were trained in a standard sampling methodology. The collector of each field sample labeled each plastic bag with their name, the time, the date, and the plot number. All samples were collected within two hours of the flight time, as in previous studies [43,44]. The plots collected after each flight/by each person were selected such that 30 of each grass type were sampled at each period, but the samples were randomized within the type. There were a total of 120 samples, and 118 after removing plots with sampling errors.

In order to best represent the vegetation measured in the aerial imagery and the vegetation most critical to fire behavior, each sample was divided into three parts. First, since most of the vegetation was dry grass, the few pieces of brush and other leafy vegetation (mostly scotch broom) were placed in a separate bag. It is unclear if a linear model, built using data primarily reflecting the much drier grass, will meaningfully reflect changes in the moisture of the sparse bushes. This moisture of this non-grass subset of the sample is referred to as “green” moisture below.

Then, the grass was separated into fuels 12” or more above the ground (denoted “top” moisture) and fuels below this threshold (denoted “bottom” moisture), using a piece of PVC pipe cut to this length as a reference. This opens up analysis to include either the total moisture or some subset of these vertically segmented parts. 

There were sixteen ground control points (GCPs), which consisted of pieces of sheet metal with black-and-white X patterns attached to the ground with stakes, throughout the study area. GPS data for the GCPs was also collected using a survey-grade GPS unit.

### 2.4. Pre-Processing Methods

The Micasense output a set of five images, each representing an individual wavelength band. The Tau outputs a video. Ffmpeg, a video processing program, was used to record the video and to extract image frames from the recorded video at 8 frames per second. 

For each of the six wavelength bands, one large orthoimage covering the study area was created. This orthorectification was performed in Agisoft Metashape using a similar processing workflow as many previous studies [45,46]. For the Micasense data, this produced only a few images that could not be aligned with no gaps in the study area. For the Tau, only a few dozen of the hundreds of images could be aligned using the default settings, and this was not improved by adjusting the settings. The images that failed initially were orthorectified by separating them into “chunks”, which could then be mosaiced together in ArcGIS Pro in a later step. Still, the preprocessed Tau data included coverage for only about 80% of the plots. For each wavelength of the Micasense data and for each chunk of the Tau data, an orthomosaic was created from a high-quality mesh.

Next, using the reflectance values for the field calibration panel, the stitched images were calibrated to the actual reflectance in each wavelength, using the calibration tool in Metashape. Then, each band was exported as an orthomosaic in.TIFF format (or in the case of the Tau, as several chunks). Next, in ArcGIS Pro, the ground control point coordinates collected in the field were used to locate one of the Micasense layers. Then, using georeferencing tools, the rest of the layers were aligned and the Tau raster was mosaiced into a single raster. The Tau “chunks” were georectified using the alignment objects in the field, and a chunk was rejected and not included in the analysis if it appeared significantly distorted or fewer than 4 alignment objects could be located.

The plot borders were digitized using the Micasense imagery and delineated everything inside the PVC border.

Outliers with very high reflectance were eliminated (set to null) to remove the influence of the PVC pipe along the plot border. By observing the values of the PVC pipes and the grass, the high reflectance threshold was estimated to be values greater than 225 for SWIR, greater than 180 for the other infrared wavelengths, and greater than 140 for the visible wavelengths. (Note that at this point reflectance is still scaled between 0 and 255 instead of 0–1). Then the mean, median, and standard deviation for the reflectance values in each wavelength were calculated for each plot. The above steps were repeated for the second flight and the summary statistics were exported in a CSV format.

A MATLAB script was created to generate image homogeneity values for each plot to serve as a second potential texture metric in addition to standard deviation.

### 2.5. Statistical Analysis

The statistical analysis was performed in R. After removing plots with sampling errors, the final sample size was *n* = 118. First, the distribution of the field sampled moisture measurements was examined. A comparison was created for different methods of calculating the moisture for the tall grass using the three subsets of moisture collected—top, bottom, and green. Top moisture alone was compared to top and bottom combined and to the total moisture of these components. The field moisture measurements were also tested for spatial autocorrelation using a Mantel test comparing two Euclidean distance matrices, one for the moistures of the three subsets of the plot and one for the coordinates of the sample locations on the 1 m sampling grid. [47]. No significant spatial autocorrelation was found (r = −0.04, *p* = 0.96).

Then, using the plot mean values for the reflectance data, the vegetation indices mentioned above (see Table 1) were calculated. For comparison, principal component analysis (PCA) was also performed on the reflectance data using the princomp function in R to create another set of predictive components. PCA uses a linear transformation to generate combinations of the input variables that are linearly uncorrelated and explain as much of the variation in the reflectance data as possible. This analysis provides an alternative way to include multiple bands in the model since the data for the various wavelengths are highly correlated with each other. PCA was performed on the five wavelengths excluding SWIR. PCA was also performed including SWIR for comparison. Multiple linear models were then created to compare the results from the commonly used vegetation indices to the principal components. Other variables were included as potential covariates: standard deviation in the NIR layer, homogeneity in the NIR layer, the time between the imagery and field data collection, the vegetation type (short or tall), and the total weight of the grass at each plot. Models were first created for each of the indices and then principal components using all potential terms. Backwards elimination was next used to eliminate parameters until there was no improvement in the model. The metrics used for assessing improvement were the Akaike information criterion and the adjusted r². For comparison, models were created for the two vegetation types separately based on the best-performing predictors for the overall models. The code used in this section as well as further details about methods are available in the Supplementary Materials.

## 3. Results

### 3.1. Comparison of Moisture Measurement Methods

**Figure 4:**
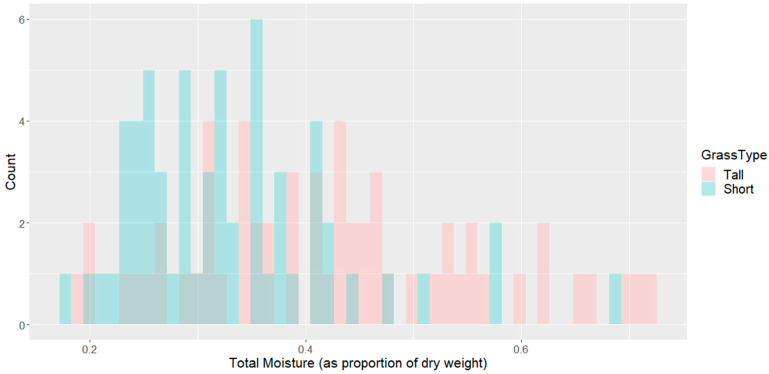
Fuel Moisture Distribution by Grass Type (Tall and Short).

The moisture distribution of the field samples is shown in Figure 4, for each of the two different vegetation types.

It is not obvious whether the reflectance in the tall grass will respond to the moisture of the entire height of the vegetation or only the moisture in the upper portion since the images are taken from above. To determine which moisture metric seemed most representative, the correlation of each with NDVI, a commonly used vegetation health metric, was compared.

**Figure 5:**
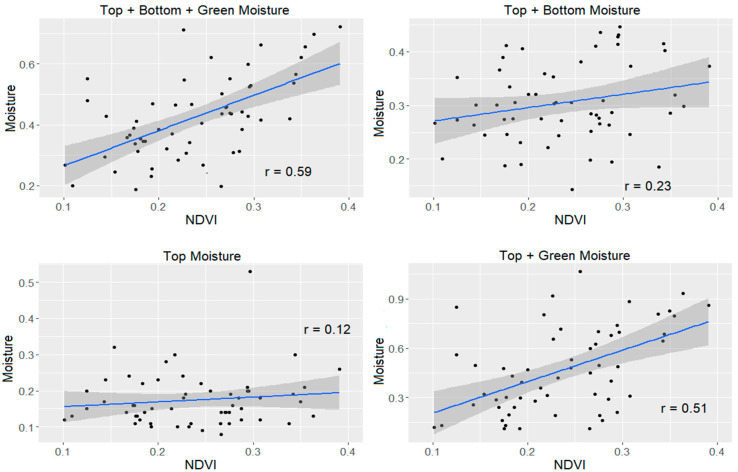
Comparison of the Relationship Between NDVI and Various Moisture Metrics for Tall Grass.

**Figure 6:**
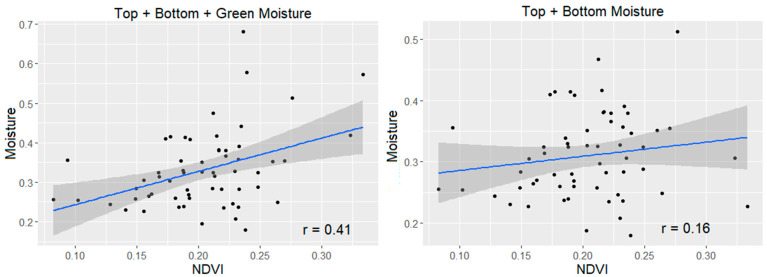
Comparison of the Relationship Between NDVI and Various Moisture Metrics for Short Grass.

For the tall grass, taking into account all of the vegetation in the plot gives the clearest relationship with NDVI, as shown in Figure 5 (upper left) where the total moisture is more correlated with NDVI than the other moisture metrics. It is also clear that the greener vegetation has a strong influence on the NDVI. Figure 6 shows that for the shorter grass, the correlation is weaker overall than the tall grass, but it is still improved by including the green moisture. For subsequent analysis, the total moisture was used.

### 3.2. Model Building

Table 3 shows PCA loadings using all the mean reflectances except the SWIR band, since the SWIR data was much less correlated with the other wavelengths than they were with each other (0.2–0.4 as opposed to 0.6–0.9 between the others).

The first four principal components are included because together they account for 99% of the variation in the reflectance data.

Table 4 summarizes the results of the regression of four different vegetation indices and principal components on field moisture measurements. The NDWI terms are not significant, but every other term is (*p* < *0*.05). NDVI, VARI, and NGR are all significantly positively correlated with moisture, but NDWI is not. The adjusted r² was included for all models by including the total weight of the field sample, a metric used to account for increased moisture in denser vegetation, which will be justified below. NDVI accounts for far more of the variation in fuel moisture than the other two significant indices based on visible wavelengths alone (VARI and NDGR both explain 17.4%).

PC1 was not a significant predictor of moisture in a model that included *all* principal components and weight, and PC3 did not substantially improve a model including all four components. PC2 + PC4 and weight performed similarly but slightly better than the model with NDVI and weight.

**Figure 7:**
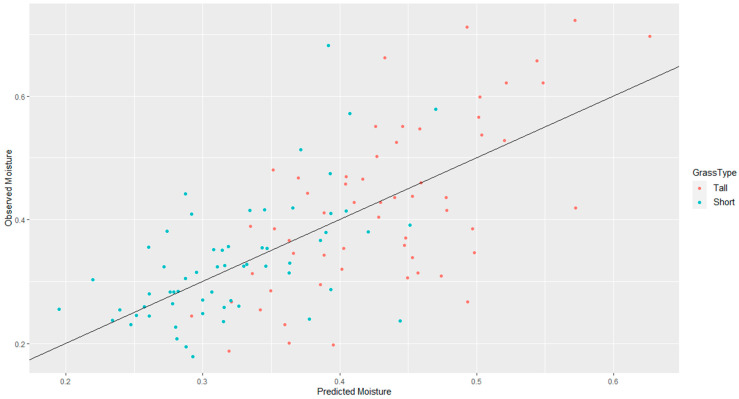
Fit for PCA + Total Weight Model by Grass Type.

Additionally, separate models were developed for the two grass types to determine if modeling each separately was more effective. A summary of a few of these is shown in Table 5. None of the models for individual grass types showed improvement over the pooled model for PC2, PC4, and total weight, likely because the sample size of each grass type is small (*n* = 59). The principal components also performed somewhat better than NDVI for the models that treated the grass types separately. The best model was therefore the pooled model that included PC2, PC4, and vegetation weight (r² = 0.45). Its AIC was also slightly lower than the NDVI model (−219 as opposed to −216). This model fit is shown in Figure 7. The equation of this model is as follows, and is discussed in the following section:

Equation (2): Equation of Best Model.

(2) LMFC= −0.061 R−0.058 B+0.057 G+0.060 RE+0.071 NIR+ 0.001 Weight+ 0.315

## 4. Discussion

### 4.1. Comparison of Models

In contrast to previous studies using MODIS data and shrublands/chapparal [30,34,38], this study does not show much predictive power for VARI and NDGR. While some research shows that VARI is a better predictor than NDVI in these other ecosystems [36], the present study agrees with many existing studies in grasslands. Others have also found that NDVI outperformed VARI in grasses, with mixed results for NDWI predictions [32,34,48]. An existing study by Lim et al. [49] developed a model using NDVI from UAV-mounted sensors to predict moisture over a similar range of values in cut hay. This study extends the relevance of this method, as much of the existing research does not focus on using UAVs to obtain data about fire fuels and instead utilizes satellite imagery or focuses on agricultural products rather than mixed vegetation. Despite the increased variation in vegetation, this model has similar performance to the Lim et al. model.

This study also shows improved model performance by using PCA instead of vegetation indices, likely because it simply includes more wavelengths. Based on the loadings in Table 3, one potential interpretation is that PC1 represents the overall brightness of the plots, a largely geometric factor that would ideally be excluded from the model. PC2 is dominated by a positive relationship between moisture and near infrared/red edge and a negative relationship with blue (based on the negative model coefficient), and PC4 represents a negative relationship with red and a positive relationship with green. PC2 could represent lower moisture content since water absorbs radiation in the region of 0.7–0.8 µm and to some degree in the blue range as well. In addition, the red edge band may be of particular importance in this study of dry grass as changes in reflectance in this band are particularly associated with senescence [28]. Therefore, it is interesting and perhaps expected that it also factors into the model. However, it is unusual that the blue wavelength is such a large part of this principal component when it tends to have only small amounts of correlation with moisture [30]. However, in this particular ecosystem, the scotch broom and some of the other greener vegetation had much lower blue reflectance than the drier grass species. PC4 provides information that intuitively would be related to fuel moisture and is similar to NDGR. Healthy vegetation strongly absorbs electromagnetic radiation in the red channel and reflects electromagnetic radiation in the green channel, resulting in positive and negative coefficients for these channels, respectively. PCA offers the advantage of including more available wavelengths in a flexible way that could easily make use of different model parameters for different ecosystems.

In the study area, especially in the short grass, there was a significant amount of exposed soil. Soil reflectance is likely to interfere with creating an accurate model in this area. Accuracy could be further improved by accounting for the effects of soil in plots that have less complete vegetation cover. Soil reflectance has been addressed in other studies with the modified soil adjusted vegetation index (MSAVI), an index which attempts to correct for varying amounts of vegetation cover [50].

### 4.2. Implications for Sensor Selection

Based on the results of this study, utilizing near-infrared sensors is useful for predicting vegetation moisture compared with using visible wavelengths alone. On the other hand, it does not provide evidence that utilizing this SWIR sensor improves the results. The range of the Tau is wider (0.9–1.7 µm) than the satellite bands often used to calculate NDWI, which are centered at 1.3 µm [28]. Some studies utilizing MODIS bands use band 5, which is 1.23–1.25 µm, to calculate NDWI and obtain modest results, though NDVI and VARI were still both better predictors in almost all cases [29,30]. The original formulation of NDWI defines it using the same wavelength as MODIS band 5 [51]. The water absorption band in this range is quite small and centered at about 1450 nm. Though the range in this study includes this wavelength, it is much larger, with a band width of 800 nm. Other effects at the lower end of the sensor range could potentially obscure the intended impacts of water absorption. A sensor with a narrower band would likely improve the ability to directly measure water thickness. Obtaining a narrow band is comparatively simple when one is making use of satellite data but less straightforward when purchasing a small UAV-mounted sensor. However, there are many readily available NIR sensors, including the one used in this study that are relatively inexpensive and user-friendly. The ability to estimate moisture using visible/NIR sensors alone is a significant management opportunity.

### 4.3. Future Improvements of Method

The results of this analysis are promising in terms of obtaining spatially explicit fuel moisture data for fire modeling. Because this is a pilot study with limited spatial and temporal scope, this study utilized vegetation with a range of moisture that was significantly smaller than other studies. Previous studies predicting moisture in grasses developed models with r ² values of 0.7–0.9, but contained 200–300% moisture ranges, instead of the less than 80% moisture range in this study [32,34]. Even some models built on vegetation that experiences less moisture content change than grass, such as shrubs or chapparal, spanned at least a 100% range of vegetation moisture [29,30]. For this reason, it is not surprising that the best model explained only a modest proportion of the vegetation moisture. In general, this study only included rather dry grass and neglected the upper part of the range of expected grass moisture values. While it is in some ways easier to obtain satellite data over a longer time frame, this problem could be addressed in future work by taking measurements throughout the fire season to develop the model and including a larger study area. In fact, early-season data can be particularly relevant for prescribed burning. In addition, while this study was too small to show clear spatial effects, research into the spatial variability of fuels is another avenue being developed that could improve this methodology [52,53]. Given these limitations, this study confirms existing research utilizing remote sensing data to estimate moisture while extending it to a different application.

In this study, the weight of the vegetation proved to be a useful predictor of vegetation moisture. Previous work shows that biomass data can be obtained using photogrammetric methods and UAV-mounted sensors, making this a promising avenue of inquiry with only a few field measurements of biomass [54]. In a 2018 study by Viljanen et al., modest to good biomass estimates were obtained for barley using only photogrammetric information and visible reflectance data [55]. Several other studies make use of UAVs and LiDAR or multispectral imagery in order to estimate aboveground biomass [56,57]. Biomass measurements can be obtained using the same sensors as the moisture measurements and are also necessary for fire modeling, so an approach like this is a promising avenue for further investigation even if these methods still require some field data for model training.

### 4.4. Future Work

The rasters generated by this methodology have more than sufficient resolution for use as an input to a model such as WFDS. Simulations at this scale use 25 cm to 1 m horizontal resolution, and this study produces rasters with 5 cm resolution. To work toward making this process operationally viable, there are many avenues for future work.

The data collection methods detailed in this paper could be improved by increasing the side overlap, sampling only representative subsets of the plots to obtain more samples closer to the time of flight, and sampling throughout the fire season to obtain a broader moisture range.

In addition, work to determine the sensitivity of fire models to the resolution and range of moisture in the input rasters would be valuable. More investigation into different ecosystems and the spatial variability of moisture, as well as its effect on fire behavior, will be necessary to develop a more broadly applicable model. Information about the relationship between fire behavior and moisture variability could also help prioritize types of ecosystems for more extensive data collection and/or modeling. In many ecosystems, larger areas could be reasonably studied with a higher flight when a lower resolution is sufficient.

Photogrammetric estimates of biomass could be used in conjunction with a method, such as the one described in this paper, to create a workflow for generating fuel data model inputs. This would allow some work toward model validation, by recording remote sensing data at a site as well as fire behavior data during a prescribed burn and comparing the fire behavior to the results of a simulation using these model inputs.

## 5. Conclusions

This method shows promise for deriving inputs to three-dimensional time-dependent fire behavior models as a way of validating fire models and improving their accuracy. Flying a UAV over an area of interest can provide data with shorter lead time and higher resolution when compared to either relying on satellite imagery or utilizing traditional field sampling methods. For example, collecting the fuel moisture for just one of the 120 1 m × 1 m sample plots in this study took approximately twice as long as a UAV flight over the entire study area. While more work is needed to make this technology operationally viable, including integrating biomass estimation, streamlining the pre-processing steps, and sensitivity analysis of the model to inform the necessary resolution of moisture information to collect, using UAV-based data collection methods could enable more accurate and current moisture data at the time of prescribed burn or other period of interest.

## Figures and Tables

**Table 1:** Common Vegetation Indices Used to Predict Moisture.

Name	Formula
NDVI	(NIR − Red)/(NIR + Red)
NDWI	(NIR − SWIR)/(NIR + SWIR)
VARI	(Green − Red)/(Green + Red − Blue)
NDGR	(Green − Red)/(Green + Red)

**Table 2:** Wavelength Bands of the Micasense (MS) and Tau Sensors, with MODIS Bands for Comparison.

Band	Wavelength Range	Wavelength of MODIS Band
Red (MS)	668 ± 7 nm	645 ± 25 nm
Green (MS)	560 ± 13.5 nm	550 ± 10 nm
Blue (MS)	475 ±16 nm	488 ± 5 nm
NIR (MS)	842 ± 28 nm	858 ± 17 nm
Red Edge (MS)	717 ± 6 nm	NA
SWIR (Tau)	1300 ± 400 nm	1240 ± 10 nm

**Table 3:** PCA Loadings with Micasense Wavelengths.

	PC1	PC2	PC3	PC4
Red	0.462	0.222	0.403	0.670
Green	0.460		0.622	−0.625
Blue	0.408	0.746	−0.474	−0.225
Red Edge	0.438	−0.317	−0.185	0.296
NIR	0.464	−0.540	−0.438	−0.152
% Variation	0.848	0.090	0.034	0.018

**Table 4:** Summary of Models Based on Vegetation Indices, PCs, and Total Sample Weight (in grams).

Index	Adj. r²	Slope	Intercept	*p*-Value
NDVI	0.329	1.189	0.115	0.000
NDWI	0.014	−0.085	0.374	0.119
VARI	0.174	0.571	0.377	0.000
NDGR	0.174	0.956	0.376	0.000
NDVI + Weight	0.431	0.930 0.001	0.093	0.000
NDWI + Weight	0.307	−0.027 0.002	0.253	0.000
VARI + Weight	0.303	0.340 0.001	0.285	0.000
NDGR + Weight	0.304	0.570 0.001	0.285	0.000
PC2	0.336	−0.124	0.380	0.000
PC2 + PC4	0.387	−0.133 −0.106	0.381	0.000
PC2 + PC4 + Weight	0.452	−0.105 −0.091 0.001	0.315	0.000
PC1 + PC2 + PC3 + PC4 +Weight	0.467	−0.004 −0.112 −0.046 −0.099 0.001	0.316	0.000

**Table 5:** Summary of Models for Separate Vegetation Types.

Index	Dominant Species	Adj. r²	Slope	Intercept	*p*-Value
NDVI	*A. elatius*	0.340	1.157	0.150	0.000
	*A. stolonifera*	0.154	0.843	0.159	0.001
VARI	*A. elatius*	0.037	0.403	0.406	0.078
	*A.stolonifera*	0.128	0.438	0.351	0.002
NDVI + Weight	*A. elatius*	0.372	1.045 0.001	0.081	0.000
	*A. stolonifera*	0.258	0.717 0.001	0.121	0.000
PC2 + PC4 + Weight	*A. elatius*	0.435	−0.141 −0.080 0.001	0.288	0.000
	*A. stolonifera*	0.316	−0.084 −0.079 0.001	0.295	0.000

## Data Availability

The data from this study can be found in the USFS data archive: Field data: https://doi.org/10.2737/RDS-2021-0070 (accessed on 22 September 2021), Micasense data: https://doi.org/10.2737/RDS-2021-0071 (accessed on 22 September 2021), Tau data: https://doi.org/10.2737/RDS-2021-0072 (accessed on 22 September 2021).

## Supplementary Materials

The following are available online at https://github.com/yesnastassia/moisture (accessed on 21 September 2021): Processing Procedures, Fieldwork Procedures, MATLAB code, R code.
